# Antagonistic TNF Receptor One-Specific Antibody (ATROSAB): Receptor Binding and In Vitro Bioactivity

**DOI:** 10.1371/journal.pone.0072156

**Published:** 2013-08-19

**Authors:** Fabian Richter, Timo Liebig, Eric Guenzi, Andreas Herrmann, Peter Scheurich, Klaus Pfizenmaier, Roland E. Kontermann

**Affiliations:** 1 Institut für Zellbiologie und Immunologie, Universität Stuttgart, Stuttgart, Germany; 2 Celonic AG, Basel, Switzerland; 3 Baliopharm AG, Basel, Switzerland; University Medical Center Freiburg, Germany

## Abstract

**Background:**

Selective inhibition of TNFR1 signaling holds the potential to greatly reduce the pro-inflammatory activity of TNF, while leaving TNFR2 untouched, thus allowing for cell survival and tissue homeostasis. ATROSAB is a humanized antagonistic anti-TNFR1 antibody developed for the treatment of inflammatory diseases.

**Methodology/Principal Findings:**

The epitope of ATROSAB resides in the N-terminal region of TNFR1 covering parts of CRD1 and CRD2. By site-directed mutagenesis, we identified Arg68 and His69 of TNFR1 as important residues for ATROSAB binding. ATROSAB inhibited binding of ^125^I-labeled TNF to HT1080 in the subnanomolar range. Furthermore, ATROSAB inhibited release of IL-6 and IL-8 from HeLa and HT1080 cells, respectively, induced by TNF or lymphotoxin alpha (LTα). Different from an agonistic antibody (Htr-9), which binds to a region close to the ATROSAB epitope but elicits strong TNFR1 activation, ATROSAB showed a negligible induction of IL-6 and IL-8 production over a broad concentration range. We further verified that ATROSAB, comprising mutations within the Fc region known to abrogate complement fixation and antibody-mediated cellular effector functions, indeed lacks binding activity for C1q, FcγRI (CD64), FcγRIIB (CD32b), and FcγRIII (CD16) disabling ADCC and CDC.

**Conlusions/Significance:**

The data corroborate ATROSAB’s unique function as a TNFR1-selective antagonist efficiently blocking both TNF and LTα action. In agreement with recent studies of TNFR1 complex formation and activation, we suggest a model of the underlying mechanism of TNFR1 inhibition by ATROSAB.

## Introduction

Tumor necrosis factor (TNF) plays an important role in the development of inflammatory diseases like rheumatoid arthritis, Crohn’s disease and the relapsing phases of multiple sclerosis. TNF is a pleiotropic cytokine that is expressed as type-II trans-membrane protein (mTNF) on the surface of macrophages, natural killer (NK) cells, B- and T-cells. It is processed into its soluble form (sTNF) by enzymatic cleavage. TNF activates two cell surface receptors, TNFR1 (CD120a) and TNFR2 (CD120b) [1], [2], [3], [4]. While TNFR1 is constitutively expressed on a broad variety of cell types, TNFR2 expression is cell type-restricted, context and stimulus-dependent and found mainly on immune cells, endothelial cells and neurons [5]. In general, stimulation of TNFR1 by sTNF or mTNF leads to pro-inflammatory and pro-apoptotic signals [6]. In contrast, effective signaling through TNFR2 is only mediated by mTNF [7], resulting in cell proliferation, tissue homeostasis and regeneration [8], [9].

Current clinical intervention in the field of inflammatory diseases is focused on the blockade of TNF, employing a soluble TNF receptor-2 fusion protein (etanercept) and anti-TNF antibodies, including infliximab, adalimumab, golimumab, and certolizumab pegol [10], [11]. Regardless of their successful clinical use, long-term treatment with TNF blockers is accompanied by a higher risk of tuberculosis (TB) reactivation and serious infections, whereas the effect of TNF blockers on incidence and/or manifestation of malignancies is discussed controversially [12], [13], [14], [15], [16]. Counterintuitive were observations that TNF blockade can be associated with development of inflammatory and autoimmune diseases [17], [18], [19], [20], indicating a highly complex regulation of TNF action in vivo.

Selective inhibition of signaling through TNFR1 holds the potential to greatly reduce the pro-inflammatory activity of TNF, while leaving TNFR2 untouched, thus allowing for cell survival, tissue homeostasis and, for the CNS, myelin regeneration [21], [22]. This change of concept in the treatment of TNF-mediated inflammatory diseases, from global ligand inhibition to selective receptor blockade, has gained increasing attention [23] and has led to the development of a number of TNFR1-selective inhibitors. For instance, the TNFR1-selective mutein R1antTNF and its PEGylated form (PEG-R1antTNF) were effectively used to treat acute hepatitis, collagen-induced arthritis (CIA), experimental autoimmune encephalomyelitis (EAE), and hyperplasia in different mouse models [24], [25], [26], [27]. A dominant-negative mutein (XENP1595) inhibits TNFR1 selectively by forming inactive complexes with sTNF and was used for the treatment of experimental colitis [28], [29], [30]. TNFR1 knockdown in mouse models by short hairpin RNA [31] and antisense oligonucleotides [32] led to the amelioration of CIA and reduced liver toxicity caused by radiation-induced TNF production. Furthermore, antibodies directed against TNFR1, such as H398 [33], [34], [35], represent another promising approach for selective TNFR1 blockage.

In previous studies we transformed a humanized Fab fragment (IZI-06.1) of H398 [36], selectively recognizing human TNFR1, into a whole IgG format [37]. This antagonistic TNF receptor one-specific antibody (ATROSAB) was shown to retain TNFR1 selectivity and to inhibit TNFR1-mediated cell responses such as cell death induction, IL-6 and IL-8 release. In addition, the kinetic constants of the binding to TNFR1 were determined using a quartz crystal microbalance (QCM) system and the epitope targeted by ATROSAB was located to the cysteine-rich domains (CRD) one and two of TNFR1 [37].

Here, we identified critical amino acids within the ATROSAB epitope of TNFR1 and studied in detail kinetic binding constants by QCM as well as functional activities in comparison to an agonistic TNFR1-specific antibody that binds an epitope in close proximity to the ATROSAB epitope. Based on these data and previous results on TNFR1-TNF signal complex formation, we suggest a model of the underlying mechanism of antibody-mediated TNFR1 inhibition and activation, respectively.

## Results

### ATROSAB Lacks Fc-mediated Effector Functions

**Figure 1:**
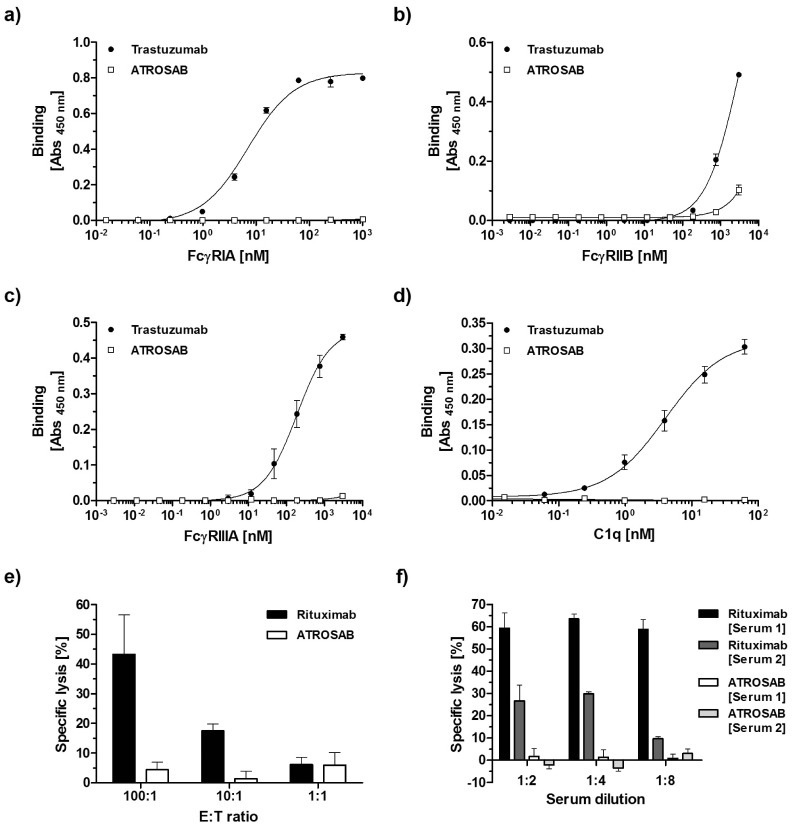
Analysis of ATROSAB for binding to human soluble Fcγ receptors and human complement protein C1q and the lack of the induction of ADCC and CDC. Binding of immobilized ATROSAB and Trastuzumab to human soluble Fcγ receptor IA (a), Fcγ receptor IIB (b), Fcγ receptor IIIA (c), and human C1q (d). e) ADCC of ATROSAB and an anti-CD20 monoclonal antibody Rituximab on Kym-1 cells and DOHH-2 cells, respectively, using different effector to target cell ratios (n = 4). f) CDC of ATROSAB and Rituximab on Kym-1 cells and DOHH-2 cells, respectively, using serum from two different donors and various serum dilutions (n = 4). Asterisks indicate statistically significant differences (** p<0.01, *** p<0.001).

The Fc region of ATROSAB is modified in order to inactivate effector functions (ADCP, ADCC and CDC) [38]. To verify that binding of Fcγ receptors and C1q is blocked in ATROSAB, binding studies with human C1q as well as human soluble Fcγ receptor IA (CD64), FcγRIIB (CD32b), and IIIA (CD16a) were performed by ELISA. ATROSAB did not bind human sFcγRIA (Fig. 1a), and sFcγRIIIA (Fig. 1c). Binding to sFcγRIIB (Fig. 1b) and C1q (Fig. 1d) was strongly reduced. In contrast, trastuzumab (possessing a human wild-type γ1 Fc) used as positive control showed binding with EC_50_ values in the described range [39], [40]. Immobilization of both antibodies was confirmed with an anti-human Fc-specific antibody (not shown).

In order to demonstrate the depletion of the ADCC effector function, the potential of ATROSAB to elicit killing of TNFR1-expressing target cells was tested with calcein-labeled Kym-1 cells. These cells express approximately 3.000 TNFR1 on the cell surface [41]. Flow cytometry studies confirmed binding of ATROSAB to Kym-1 (data not shown). Rituximab, a chimeric anti-CD20 IgG1, and DOHH-2 cells were included as positive control. As expected, the control antibody induced lysis of target cells by ADCC depending on the E:T cell ratio (Fig. 1e). In contrast, ATROSAB induced-cell lysis of Kym-1 cells was significantly reduced when added at 600 µg/ml even at a high E:T ratio (Fig. 1e). Similar results were obtained with lower ATROSAB concentrations (120 µg/ml and further 1∶5 dilutions), whereas lower concentrations of rituximab were still able to elicit ADCC in DOHH-2 cells, particularly at the highest E:T ratio (not shown).

The potential of ATROSAB to elicit complement-dependent cytolysis of target cells (CDC) was also tested employing calcein-labeled Kym-1 as target cells. Again, Rituxumab and CD20-expressing DOHH-2 cells served as positive control. As expected, the positive control antibody led to cytolysis of the target cells using serum from two different donors (Fig. 1f). In contrast, ATROSAB elicited significantly reduced CDC in antigen-expressing Kym-1 cells (Fig. 1f). Similar results were obtained with lower ATROSAB concentrations (60 µg/ml and further dilutions), whereas lower concentrations of rituximab were still able to elicit CDC in DOHH-2 cells (not shown). Taken together, these data confirm that ATROSAB does not induce a substantial CDC and ADCC activity against TNFR-1 expressing target cells.

### Receptor Binding and TNF Blocking of ATROSAB

**Figure 2:**
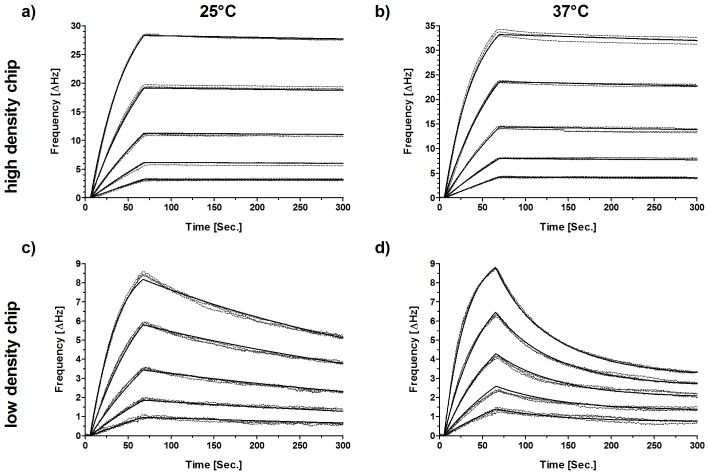
QCM analysis of human TNFR1 binding by ATROSAB. Binding of ATROSAB to human TNFR1 was analyzed by QCM at high (a, b; 130 Hz) and low (c, d; 27 Hz) density of immobilized human TNFR1-Fc. ATROSAB was analyzed at concentrations between 2–32 nM at 25°C (a, c) and 37°C (b, d) in triplicates for each concentration (dashed lines = data curves, solid lines = fitted curves).

Binding of ATROSAB to human TNFR1 was analyzed by quartz crystal microbalance (QCM) measurements at high and low densities of immobilized receptor (Fig. 2a, c). At room temperature and a high receptor density, ATROSAB bound with high avidity revealing an apparent dissociation constant (K_d_ value) of 0.12 nM. At lower receptor density, a reduced binding (K_d_ = 1.7 nM) was observed (Table 1). In addition, binding studies were performed at 37°C to determine binding at physiological temperature (Fig. 2b, d). At high receptor density only minor differences of the kinetic constants were observed compared to 25°C (K_d_ value of 0.19 nM) (Table 1). However, at lower receptor density the curves obtained at 37°C could only be fitted assuming a biphasic, i.e. mono- and bivalent binding of ATROSAB to immobilized TNFR1. The subtraction of the slowly dissociating part (obtained at high receptor density) yielded an apparent K_d_ value of 14.2 nM for the monovalent interaction, which resulted from a faster off-rate (Fig. 2d, Table 1).

**Table 1:** QCM affinity measurements of ATROSAB.

receptor density	T (°C)	k_on_ (M^−1^s^−1^)	k_off_ (s^−1^)	K_D_ (nM)
130 Hz	25	7.4×10^5^	9.2×10^−5^	0.12
130 Hz	37	8.8×10^5^	1.6×10^−4^	0.19
27 Hz	25	1.4×10^6^	2.4×10^−3^	1.68
27 Hz	37	1.6×10^6^	2.3×10^−2^	14.2#

# these data refer to the monovalent affinity interaction at low receptor density.

**Figure 3:**
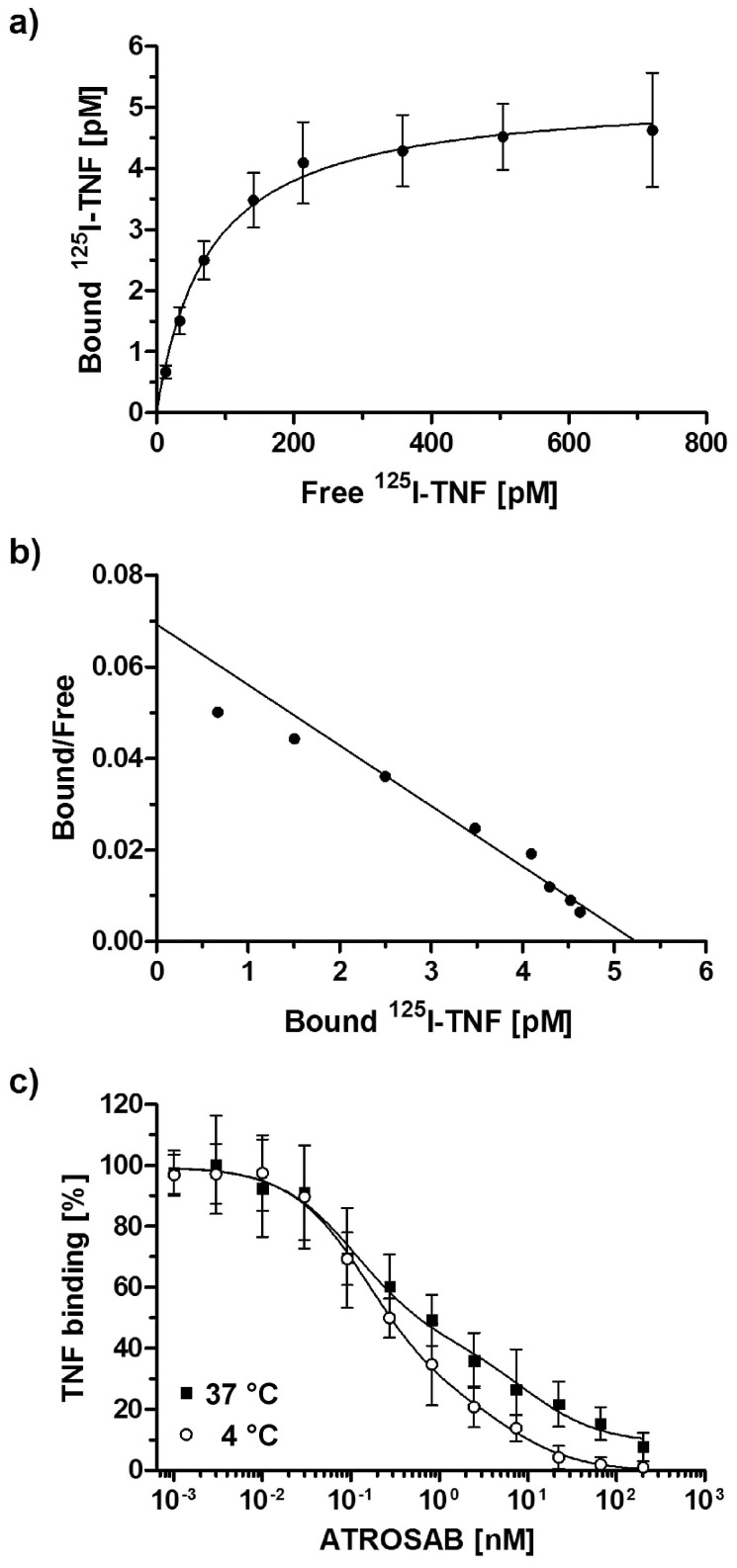
Inhibition of ^125^I-TNF binding to HT1080 cells by ATROSAB. a) Binding of ^125^I labeled TNF to HT1080 cells. b) Scatchard blot of binding of ^125^I labeled TNF to HT1080 cells. c) Inhibition of binding of ^125^I labeled TNF to HT1080 cells by ATROSAB at 4°C and 37°C, respectively. Displayed are mean values and SD of three individual experiments in percent of maximal TNF binding.

Next, we investigated the inhibitory activity of ATROSAB on TNF binding to HT1080 cells using ^125^I-labeled TNF. Approximately 1500 binding sites for ^125^I-labeled TNF were determined on HT1080 cells from a saturation binding curve (Fig. 3a). ^125^I-labeled TNF bound to HT1080 cells with a K_d_ value of 0.11 nM (Fig. 3b). Binding of ^125^I-labeled TNF at a concentration of 0.1 nM was inhibited by ATROSAB in a concentration-dependent manner. Analysis of the inhibition curves obtained at 4°C and 37°C revealed a two-site inhibition mode, with IC_50_ values of 0.11 nM and 9.58 nM at 4°C. Similar values were determined at 37°C (IC_50_ values of 0.15 nM and 6.01 nM) (Fig. 3c). These results confirm that ATROSAB exhibits its inhibitory activity directly by blocking the binding of TNF to its receptor.

### Inhibition of IL-8 and IL-6 Release Induced by TNF and Lymphotoxin Alpha (LTα)

**Figure 4:**
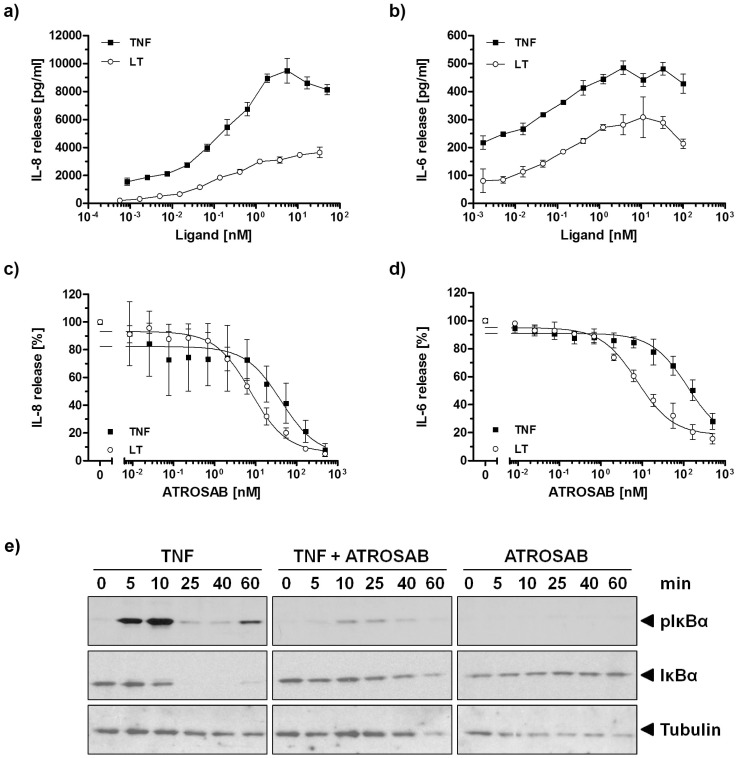
Inhibition of TNF and LTα action by ATROSAB. a) IL-8 release by HT1080 cells triggered by TNF and LTα b) IL-6 release by HeLa cells triggered by TNF and LTα. c) Inhibition of TNF- and LTα-induced IL-8 secretion by HT1080 cells with increasing concentrations of ATROSAB using 0.1 nM of TNF and LTα. d) Inhibition of TNF- and LTα-induced IL-6 secretion by HeLa cells with increasing concentrations of ATROSAB using 0.1 nM of TNF and LTα. Data from n = 3 experiments are shown as percent of maximal binding (c, d), shown are mean values and standard deviation. e) Immunoblot analysis of inhibition of TNF-induced phosphorylation (pIκBα) and degradation of IκBα in HT1080 cells by excess amounts of ATROSAB. Tubulin was included as loading control.

We analyzed the neutralizing activity of ATROSAB on IL-8 and IL-6 secretion induced by lymphotoxin alpha (LTα, LTα_3_) in comparison to TNF-induced cytokine release. With the reagents used in the HT1080 cell model, LTα-induced IL-8 reached approximately 40% of the maximum value induced by TNF (Fig. 4a). Similarly, LTα-induced release of IL-6 from HeLa cells reached approximately 65% of the maximum value induced by TNF (Fig. 4b). ATROSAB inhibited IL-8 and IL-6 secretion induced by 0.1 nM LTα (5.7 ng/ml) with an IC_50_ value of 7.6 nM, which proved to be more efficient than blocking TNF (0.1 nM) induced IL6 and IL8 secretion in these in vitro models (Fig. 4c and d).

Furthermore, we analyzed the effects of ATROSAB on TNF-mediated signal transduction as determined by phosphorylation of IκBα. HT1080 cells incubated with TNF (0.1 nM) induced rapid phosphorylation of IκBα and subsequent degradation as shown by immunoblotting experiments with anti-IκBα and anti-pIκBα antibodies (Fig. 4e). In the presence of excess amounts of ATROSAB, phosphorylation and degradation of IκBα was strongly reduced, demonstrating that ATROSAB inhibits TNF-induced signal transduction. ATROSAB alone did not induce any IκBα phosphorylation.

### Comparison of ATROSAB with the Agonistic Antibody Htr-9

**Figure 5:**
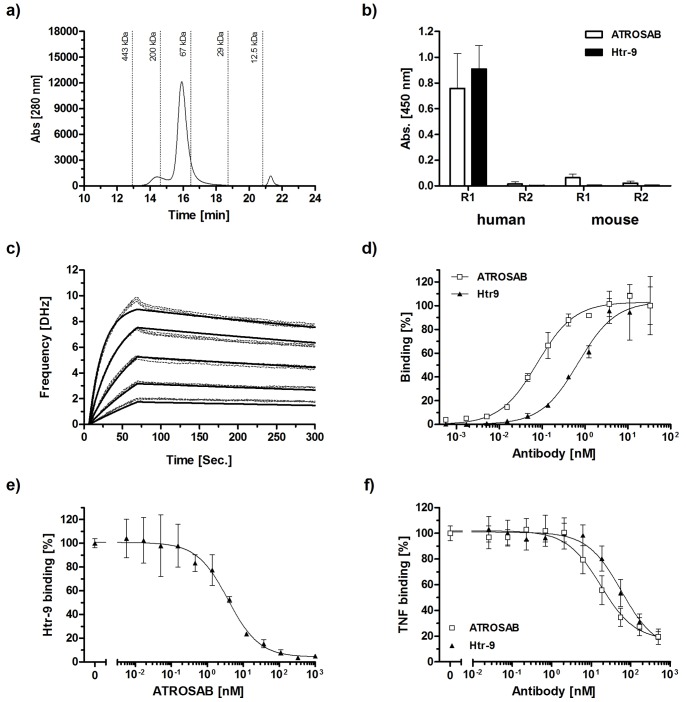
Characterization of the agonistic antibody Htr-9. a) SEC of purified Htr-9. b) ELISA analyzing binding of Htr-9 and ATROSAB to immobilized human and mouse TNFR1-Fc and TNFR2-Fc. c) Binding of Htr-9 to human TNFR1-Fc analyzed by QCM at a medium receptor density (indicated by a 48 Hz resonance frequency shift). Htr-9 was analyzed at concentrations between 1 µM–62.5 nM. d) Binding of ATROSAB and Htr-9 to immobilized human TNFR1-Fc in ELISA. e) Inhibition of binding of Htr-9 (7 nM) to human TNFR1-Fc by increasing concentrations of ATROSAB. f) Inhibition of binding of TNF (1 nM) to immobilized human TNFR1-Fc in ELISA by increasing concentrations of Htr-9 or ATROSAB, respectively.

Monoclonal antibody Htr-9 is described as a human TNFR1 agonist [42], [43]. Purified Htr-9 showed in size exclusion chromatography (SEC) a major peak corresponding to intact IgG (Fig. 5a). Htr-9 bound human TNFR1 in ELISA while no binding was seen for human TNFR2 and mouse TNFR1 and TNFR2. The same results were obtained for ATROSAB (Fig. 5b) demonstrating that both antibodies are specific for human TNFR1. By QCM, a K_d_ value of 14 nM was determined for human TNFR1-Fc at a receptor density of 48 Hz (Fig. 5c). In ELISA, Htr-9 showed strong binding to immobilized TNFR1-Fc, although binding was weaker than that seen for ATROSAB (EC_50_ values: 0.08 nM for ATROSAB and 0.7 nM for Htr-9) (Fig. 5d). ATROSAB competed with Htr-9 for binding to human TNFR1-Fc (IC_50_ = 3.8 nM), indicating that the two antibodies share overlapping epitopes (Fig. 5e). Htr-9 inhibited binding of TNF to TNFR1-Fc in ELISA with an IC_50_ value of 58 nM. ATROSAB, included as control, showed inhibition of TNF binding with an IC_50_ value of 17 nM (Fig. 5f).

**Figure 6:**
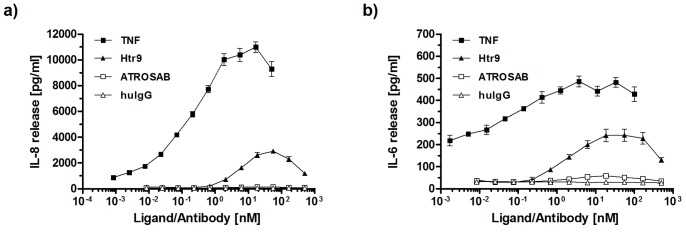
Analysis of cytokine release, induced by TNF, Htr-9, and ATROSAB. Effects of increasing concentrations of TNF, Htr-9 and ATROSAB on release of IL-8 from HT1080 (a) and and IL-6 from HeLa cells (b). Human serum IgG was included as negative control. Cells were incubated for 16 h with the proteins and released cytokines were determined by ELISA. Mean values and standard deviation of three independent experiments are shown.

Next, we analyzed ATROSAB and Htr-9 for their potential to induce cytokine secretion in vitro. For comparison, we included human TNF, which induced a strong release of IL-6 from HeLa cells and IL-8 from HT1080 cells, respectively (Fig. 6). Maximum cytokine release was observed at a TNF concentration of approximately 10 nM, which resulted in IL-8 concentrations of 6–19 ng/ml and IL-6 concentrations of 300–700 pg/ml in the cell culture supernatant. Htr-9 showed a clear agonistic activity leading to strong IL-6 and IL-8 secretion (with a maximum of 1–5 ng/ml for IL-8 and 200–300 pg/ml for IL-6 at around 30 nM of Htr-9), corresponding to approximately 26% and 50% of the maximum TNF activity, respectively (Fig. 6). In contrast, ATROSAB led only to a marginal cytokine release reaching a maximum of 120–220 pg/ml IL-8 and of 40–80 pg/ml IL-6 (background values of cells alone: 50–70 pg/ml IL-8 and 25–40 pg/ml IL-6). This weak activity corresponds to approximately 1% and 5% of the in vitro TNF activity detected in IL-8 release (HT1080) and IL-6 release (HeLa) assays, respectively. Control IgG had no discernible effect on cytokine secretion (Fig. 6).

### Epitope Mapping

**Figure 7:**
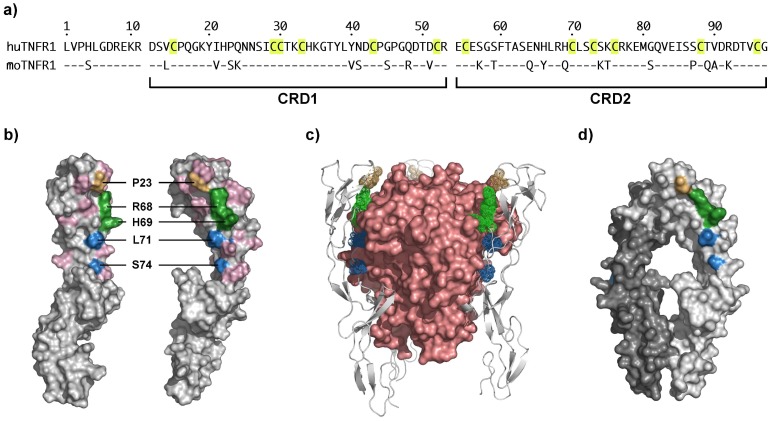
Mapping of the Epitope of ATROSAB on human TNFR1. a) Comparison of amino acids 1–97 of human and mouse TNFR1 b). Positions of the identified residues involved in binding of ATROSAB (orange, green) and Htr-9 (blue) are highlighted. c) Location of the identified residues in the complex of LTα and TNFR1 (pdb entry 1TNF; [44]), colors as in b). d) Location of the identified residues in the TNFR1 dimer (pdb entry 1FT4; [65]), colors as in b). Structures were visualized using Pymol (The PyMOL Molecular Graphics System, Version 1504 Schrödinger, LLC).

In order to map the epitope of ATROSAB and Htr-9, we generated a panel of mutant human TNFR1-Fc fusion proteins carrying mutations of one or two residues substituting the residue(s) either with those from mouse TNFR1 (Fig. 7a, Table 2) or with alanine. All proteins were produced in stably or transiently transfected HEK293 cells and purified by protein A chromatography. Purity of all fusion proteins was confirmed by SDS-PAGE (data not shown). Binding of ATROSAB as well as human TNF to immobilized receptor-Fc fusion proteins was analyzed by ELISA using 1 nM ATROSAB or TNF. Data were standardized towards coating control (anti-human IgG Fc-specific antibody). Under these conditions, a strongly reduced binding of ATROSAB was seen with mutants P23S, R68A, H69Q and H69A, while all mutants showed binding of TNF (Table 2). In a previous study, we found that the double mutation P23S/Q24K in a chimeric human/mouse TNFR1 completely abrogated ATROSAB binding [37]. This was narrowed down to a single amino acid using the same mutations in the fully human TNFR1 background. Single mutations of P23 or Q24 to either the mouse residue or an alanine revealed that P23 is critical for ATROSAB binding, while mutation of Q24 to lysine or alanine did not affect binding. Furthermore, residue H69 (mutated to Q or A) contributes also strongly to binding of ATROSAB. Binding of ATROSAB and TNF to selected mutants was further analyzed by titration experiments (Table 3). The wild-type receptor bound ATROSAB and TNF in the subnanomolar range. Mutation of H69 to alanine reduced binding of ATROSAB approximately 440-fold (Table 3). An 18-fold reduction was observed for the mutation of the conserved residue R68 to alanine. All mutants bound TNF with similar EC_50_ values as wild-type TNFR1 (Table 3). Epitope fine mapping was also performed for the described TNFR1-agonistic antibody Htr-9. Using human-mouse chimeric TNFR1 molecules [37], the epitope of Htr-9 was located between residues 29 and 137 (B2 of CRD1, CRD2 and CRD3) (not shown). Using TNFR1 mutants, two residues (L71 and S74) were found to be part of the Htr-9 epitope, discriminating the epitope of ATROSAB from that of the agonistic antibody Htr-9 (Fig. 7b, Table 2). A structural visualization in the LT-TNFR1 complex (pdb entry 1TNR; [44]) showed that the epitopes of both antibodies are located at the ligand-binding site of the receptor (Fig. 7b and 7c).

**Table 2:** Epitope fine mapping of ATROSAB analyzed by binding to TNFR1 mutants in ELISA.

		Binding		
Receptor	mutation	TNF	ATROSAB	Htr-9
moTNFR1	wt	+	−	−
huTNFR1	wt	+	+	+
huTNFR1	V14L	+	+	+
huTNFR1	I21V	+	+	+
huTNFR1	P23S	+	−	+
huTNFR1	Q24K	+	+	+
huTNFR1	G45S	+	+	+
huTNFR1	Q48R	+	+	+
huTNFR1	D51V	+	+	+
huTNFR1	S57K	+	+	+
huTNFR1	S59T	+	+	+
huTNFR1	E64Q	+	+	+
huTNFR1	H66Y	+	+	+
huTNFR1	R68A	+	−	+
huTNFR1	H69Q	+	−	+
huTNFR1	H69A	+	−	+
huTNFR1	L71A	+	+	−
huTNFR1	S72A	+	+	+
huTNFR1	S74K	+	+	−
huTNFR1	K75T	+	+	+
huTNFR1	G81S	+	+	+
huTNFR1	S87P	+	+	+
huTNFR1	T89Q/V90A	+	+	+
huTNFR1	R92K	+	+	+

binding to the receptor mutants compared to huTNFR1-Fc is indicated as similar (+) or strongly reduced (−), respectively. Receptors were immobilized at 1 µg/ml and incubated with antibodies or TNF at a concentration of 1 nM.

**Table 3:** Binding of ATROSAB and TNF to human TNFR1 mutants in ELISA.

TNFR1	ATROSAB binding	TNF binding
Mutant	EC_50_ (nM)	EC_50_ (nM)
Wt	0.27	0.19
P23S	11.0	0.16
Q24K	0.23	0.17
H66Y	0.18	0.14
R68A	5.0	0.16
H69Q	8.6	0.15
H69A	118.5	0.14
R92K	0.12	0.08

Details of binding studies are described in Materials and Methods.

## Discussion

ATROSAB is a humanized IgG1 antibody with a mutated heavy chain described to prevent the induction of CDC and ADCC [38]. Here, we could show that ATROSAB indeed lacks binding to C1q and FcγRIA and does not induce ADCC or CDC in TNFR1-expressing cells. Of note, ATROSAB shows strongly reduced binding to FcγRIIIA and the inhibitory FcγRIIB. Notably, co-engagement with FcγRIIB was shown to be required for *in vivo* activity of agonistic antibodies, such as antibodies directed against members of the TNF receptor superfamily [45]. The lack of binding of ATROSAB to FcγRIIB should, therefore, prevent co-engagement of this receptor with ATROSAB avoiding undesired agonistic activities.

ATROSAB binds to human TNFR1 and is thereby capable of inhibiting binding of the TNFR1 ligands, TNF and LTα. Inhibition of binding of ^125^I-labeled TNF to HT1080 cells revealed a two-site competition curve, indicating mono- and bivalent interactions of ATROSAB with membrane-expressed TNFR1. This was confirmed by QCM measurements using low and high densities of surface-immobilized TNFR1-Fc. Here, the calculated apparent K_d_ values of 14 nM for binding at low receptor density and 0.2 nM at high receptor density point to avidity effects due to mono- and divalent binding of ATROSAB, which is in accordance with similar effects described for other IgG and IgM molecules [46], [47]. The reduced binding of ATROSAB at a low receptor density was mainly caused by a faster off-rate, which was particularly apparent at physiological temperature (37°C).

**Figure 8:**
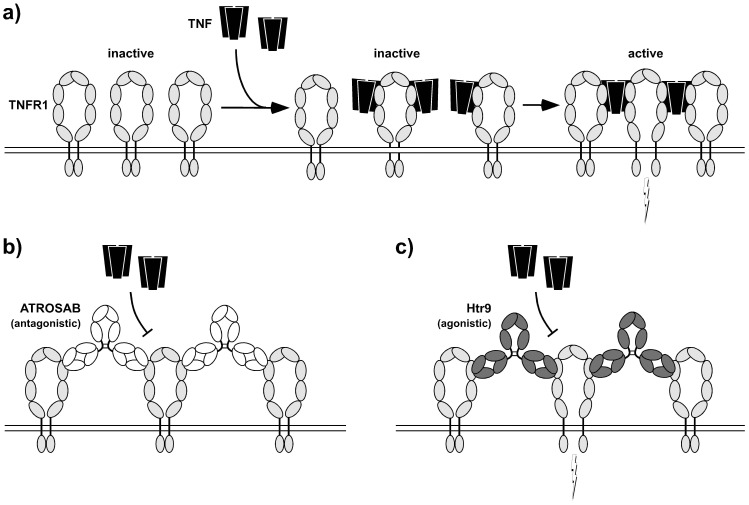
A model for TNF-TNFR1 interaction, activation and inhibition. a) Proposed model of binding of TNF to TNFR1 and formation of signaling complexes. Binding of TNF to two or more TNF receptors leads to a conformational change and activation of signal transduction [56]. b) ATROSAB binding to TNFR1 results in formation of receptor-antibody complexes, however, does not lead to a conformational change in the receptor. c) Binding of agonistic antibody Htr-9 mimics activation by TNF leading to a conformational change of the receptor.

ATROSAB inhibited TNF- and LTα-induced IL-6 and IL-8 release from HeLa and HT1080 cells, respectively, which could be fitted by an one-site competition curve. Compared to TNF, activity of LTα was inhibited more effectively by ATROSAB, most likely due to the lower affinity of LTα for TNFR1 [48]. Interestingly, IL-6 and IL-8 release was not affected by ATROSAB at concentrations where ^125^I-TNF binding was already strongly reduced (0.01–1 nM; compare Fig. 3 and 4). This finding indicates that activation of only a few TNFR1 is sufficient to induce a cellular response. Inhibition of TNFR1 signaling complexes by ATROSAB may be affected by different populations of monomeric and preassembled multimeric TNFR1 [49] and/or the inhomogeneous distribution and motility of TNFR1 in membrane microdomains [50], [51], [52]. Moreover, the different affinities of TNF for monomeric and dimeric TNFR1 molecules (P.S., unpublished data) may influence binding of ATROSAB and inhibition of the formation of TNFR1 signaling complexes.Contrary to a long-standing dogma, recent data support the assumption that pre-ligand-binding assembly domain (PLAD)-associated receptor dimers represent the smallest signaling unit, rather than a trimeric receptor assembly [53]. As shown by computational simulation of TNF-TNFR1 interactions, binding of TNF to a TNFR1 homodimer may abrogate the PLAD-mediated receptor-receptor interaction leading to initial complexes where a single TNF homotrimer is bound to two TNFR1 molecules [54]. These initial complexes can then form larger clusters [55] via newly formed PLAD-PLAD interactions. Notably, in this model, binding of two PLAD-linked receptors to two distinct TNF homotrimers engaged in these clusters is sufficient for receptor activation. This is in excellent agreement with a recent modeling study [56], proposing that active TNFR1 signaling units are receptor dimers conformationally switched “ON” by ligand binding (see also Fig. 8), while unligated TNFR1 homodimers seem to be mainly in an “OFF” conformation. Key differences between the ON and the OFF status might be the distances between the intracellular death domains [56], [57], [58], allowing efficient TRADD association only in the ON status. Interestingly, the data by Lewis et al. suggest that TNF interaction *per se* does not induce a conformational change in the receptor domains. TNF rather stabilizes the PLAD-linked extracellular domains in ON conformation when bound to two receptors simultaneously, such that the increased distance of the two adjacent intracellular domains allows TRADD binding. Accordingly, we propose that only TNF molecules linked to at least two receptors, i.e. integrated in larger clusters, activate their bound receptors. In line with this model, antibodies might be capable of stabilizing either the ON or the OFF conformation, depending on the particular location or geometry of the epitope. Agonistic anti-TNFR1 antibodies, such as Htr-9, mimic binding of TNF to TNFR1 shifting the equilibrium towards the active conformation. In contrast, antagonistic antibodies, such as ATROSAB, inhibit ligand binding and keep the bound receptors mainly in the inactive conformation (Fig. 8). Interestingly, agonistic Htr-9 and antagonistic ATROSAB share an overlapping epitope as shown by competition ELISA and mutational analysis, both epitopes located within the ligand-binding site of TNFR1. These findings indicate that subtle differences in binding of a bivalent IgG molecule can be responsible for converting TNFR1 into an active signaling complex and that sole receptor dimerization by antibodies is insufficient for activation.

In summary, we provide further evidence and a rational for the unique properties of ATROSAB, a promising new antibody for the treatment of inflammatory and neurodegenerative diseases. ATROSAB is a bivalent IgG1 that binds with high avidity to TNFR1 without inducing significant receptor activation, in contrast to other TNFR1-binding antibodies, which show a strong agonistic activity. Different from TNF blocking antibodies, ATROSAB efficiently blocks binding of both, TNF and LTα to TNFR1-expressing cells. Growing evidence supports a proinflammatory role of LTα [59], e.g. shown in studies of collagen-induced arthritis (CIA) and experimental autoimmune encephalomyelitis (EAE) [60], [61], [62]. Resistance to infliximab treatment in RA patients could be circumvented with etanercept, a TNFR2-Fc fusion protein also blocking LTα, indicating that LTα plays an important, in some patients apparently a dominant role in this disease [63]. Different to the five approved biologics targeting the TNF pathway, ATROSAB binds selectively to TNFR1, thus blocking all proinflammatory signals mediated by this receptor, while keeping TNFR2, involved in cell proliferation, tissue homeostasis and regeneration, totally unaffected. In conclusion, ATROSAB blocks the activity of TNF and LTα on TNFR1, thus, combines the beneficial effects of selective TNFR1 blockade with the advantages seen for inhibition of TNF and LTα activity.

## Materials and Methods

### Antibodies and Proteins

ATROSAB was provided by Baliopharm (Basel, Switzerland). Htr-9 was a generous gift of Dr M. Brockhaus (Hoffmann-La Roche, Basel, Switzerland). Anti human IgG-PE was purchased from Sigma-Aldrich. Trastuzumab was kindly provided by Prof. Heidtmann (St. Joseph Hospital, Bremerhaven, Germany) and Rituximab (MabThera) was obtained from Roche (Germany). Recombinant human TNF (2×10^7^ units/mg) and lymphotoxin alpha (LTα; 6×10^7^ units/mg) were provided by Knoll (Ludwigshafen, Germany) and Bender MedSystems (Vienna, Austria). IL-6 and IL-8 ELISA kits were purchased from Immunotools. Anti-phospho-IκBα (Ser32/36-specific) (5A5) mouse mAb #9246 and anti-IκBα (L35A5) mouse mAb (amino-terminal antigen) #4814 were purchased from Cell Signaling (Frankfurt, Germany). Anti-tubulin mAb MS-581-P1 was purchased from Neomarkers (Fremont, USA) and HRP-conjugated anti-mouse IgG (Fc specific; A 2554) was purchased from Sigma-Aldrich. C1q was purchased from Calbiochem (Darmstadt, Germany). Recombinant soluble human FcγRI (CD64; aa 1–288), FcγRIIB (CD32b; aa 46–217), and FcγRIII (CD16a; aa 1–208) were purchased from Hölzel Diagnostika (Köln, Germany).

### Production of TNFR1 Mutants

TNFR1 mutants were generated by site directed mutagenesis using standard PCR techniques. HEK293 cells were transfected by lipofection (Oligofectamine™ Reagent, Invitrogen, Carlsbad, USA) and proteins were purified from the supernatant by protein A affinity chromatography (TOYOPEARL® AF-rProtein A-650F, Tosoh, Stuttgart, Germany).

### Affinity Measurements

Affinities of ATROSAB and Htr-9 for human TNFR1-Fc were determined by quartz crystal microbalance measurements (Attana A-100 C-Fast system). Human TNFR1-Fc was chemically immobilized on a carboxyl sensor chip according to the manufacturer’s protocol at a high and low density, respectively. Binding experiments were performed in PBST (PBS, 0.1% Tween 20) pH 7.4 with a flow rate of 25 µl/min at 25°C and 37°C. The chip was regenerated with 25 µl 5 mM NaOH. Before each measurement, a baseline was measured which was subtracted from the binding curve. Data were collected by Attester 3.0 (Version 3.1.1.8, Attana, Stockholm, Sweden) and analyzed by Attache Office Evaluation Software (Version 3.3.4, Attana, Stockholm, Sweden), using a simple or a mass transport model for curve fitting. To analyze the biphasic kinetics detected at 37°C and the low receptor density (27 Hz), we subtracted a set of curves, generated by using the kinetic constants measured at 37°C and the high receptor density (130 Hz) representing bivalent binding, and aligned them to the last time point of the original dataset individually for each concentration. The residual curves showed reasonable fitting to a one site binding model. The superimposition of both fits described the original data almost exactly.

### Competition of ^125^I-labeled TNF

TNF was labeled with Na^125^I (Hartmann Analytic GmbH) using iodination beads (IODO-BEADS Iodination Reagent, Thermo Scientific) to specific activities of 1–2×10^4^ cpm/ng. The retained biologic activity (40–60%) was determined in cytotoxicity assays with Kym-1 cells [64]. The iodination beads were washed with 1 ml PBS and two of the beads were pre-incubated with 10 µl Na^125^I and 50 µl PBA for 5 min at RT. 10 µg TNF was incubated for 4–10 min with iodination beads and Na^125^I. The supernatant was applied to a PD-10 column (Sephadex™ G-25 M, GE Healthcare) and eluted with 10×1 ml PBS. Eluted fractions were analyzed in a gamma counter (Berthold, Wildbad, Germany) and the 2–3 main fractions were pooled. The final concentration of ^125^I-TNF was adjusted to 2 ng/µl. For competition assays, HT1080 cells were detached, resuspended in a PBA suspension (PBS, 1% BSA, 0.1% NaN_3_) and 2×10^5^ cells were seeded per well. 200 nM ATROSAB was titrated in dilution steps of 1∶3 to a constant concentration of 0.1 nM ^125^I-TNF. Non-specific binding was determined using a 200-fold molar excess of unlabeled TNF. 0.1 nM ^125^I-TNF alone served as 100% binding control. The samples were incubated for 3 hours at 4°C or 37°C. Cells and supernatant were separated by centrifugation at 13,200 rpm in micro-tubes (Sarstedt) containing 150 µl of an oil mixture (dibutylphtalate/dioctylphtalate) with a density of 1.014 g/cm^3^ (adjusted to let the cells settle to the ground, separated from the aqueous phase on top of the oil). The binding of ^125^I-TNF was analyzed in a gamma counter (Berthold, Wildbad, Germany). The inhibition of TNF binding by ATROSAB was analyzed using a two-site competition model.

### Cytokine Release

2×10^4^ HeLa or HT1080 cells per well were seeded into a 96 well microtiter plate and grown in 100 µl RPMI 1640, 5% FCS overnight. The next day, the supernatants were exchanged in order to remove constitutively produced IL-8. The cells were incubated with dilution series of TNF and LTα (100 nM–1.7 pM in RPMI 1640, 5% FCS) at 37°C for stimulation experiments (unstimulated cells served as control). For TNF and LTα inhibition experiments, the cells were incubated with dilution series of ATROSAB (500 nM–8.4 pM in RPMI 1640, 5% FCS) at 37°C, in the presence of 0.1 nM TNF or LTα (TNF or LTα alone served as controls). After 16 hours, the plates were centrifuged at 1,500 g for 5 minutes. The cell supernatants were analyzed directly by ELISA using the high sensitivity human ELISA Set for IL-6 and IL-8 (ImmunoTools).

### IL-6 and IL-8 ELISA

100 µl anti-human IL-8 or IL-6 antibody diluted 1∶100 in PBS were coated onto microtiter plates and incubated at 4°C overnight. The residual binding sites were blocked with 3% bovine serum albumin (BSA) in PBS at room temperature for 1 hour. Subsequently, the plates were incubated with 100 µl of each, the cell supernatants, diluted 1∶2 to 1∶75 in RPMI 1640, 5% FCS or the IL-8 or IL-6 standard (100 to 300 pg/ml titrated by stepwise dilution of 1∶3) and biotinylated anti-human IL-8 or IL-6 antibody and polystreptavidin-HRP conjugate (ImmunoTools, diluted 1∶15,000 in PBS, 1% BSA, 0.05% Tween20). For detection, 100 µl TMB substrate solution (3,5,3′,5′-tetramethylbenzidine) were administered to each well, the reaction was stopped by the addition of 50 µl 1 M H_2_SO_4_ and the absorption at the wavelength of 450 nm was measured using the Infinite microtiter plate reader (Tecan I control). Prior to each step, the plates were washed three times with PBS, containing 0.005% Tween20 and twice with PBS.

### IκBα Immunoblot

One million HT1080 cells in 2 ml RPMI 1640, 5% FCS were seeded in a 6-well plate one day prior to the assay. Cells were then stimulated with TNF (0.1 nM), ATROSAB (20 nM), or TNF (0.1 nM) together with ATROSAB (500 nM), respecctively, for 0, 5, 10, 25, 30 and 60 min at 37°C, 5% CO_2_. Subsequently, the supernatants were replaced by 1 ml ice cold PBS and the cells were detached mechanically. After centrifugation (500 g, 4°C, 5 min), the pellets were resuspended in “Solubi Shu” lysis buffer (150 mM NaCl, 1 mM EDTA, 20 mM Tris, 1% Triton-X-100, pH 7.6) and incubated on ice for 30 minutes. Cell debris was separated by centrifugation (16.000 g, 4°C, 10 min) and the total protein content was determined by Bradford assay. 40 µg of the total protein was analyzed by SDS-PAGE and transferred to a nitrocellulose membrane using a semidry-blotter. Residual binding sites were blocked with PBS, 5% skimmed milk. Phospho-specific mouse monoclonal antibody (mAb) for pIκBα, mouse mAb for total IκBα, mouse mAb for tubulin-α (loading control), and HRP-conjugated rabbit anti-mouse IgG (Fc-specific) antibody were used for detecting the respective protein species. Between each detection step, the membranes were stripped (5 min incubated with ddH_2_O, 5 min 0.2 M NaOH, 5 min dd H_2_O) and blocked again with PBS, 5% skimmed milk. Signals were detected with ECL substrate solution (incubated for 2 min).

### Size Exclusion Chromatography

Size exclusion chromatography (SEC) was performed by HPLC using a BioSuiteTM 250, 5 µm HR SEC (Waters GmbH, Eschborn, Germany). The following standard proteins were used: apoferritin (443 kDa), β-amylase (200 kDa), bovine serum albumin (67 kDa), carbonic anhydrase (29 kDa), aprotinin (6.5 kDa).

### ELISA

Microtiter plates were coated with 100 µl of human TNFR1-Fc fusion protein [37] at 1 µg/ml in PBS and incubated at 4°C overnight. The residual binding sites were blocked with MPBS (2% skim milk in PBS, 200 µl per well) at room temperature for 2 hours. 100 µl ATROSAB or Htr-9 (100 nM–5.7 pM, diluted in 2% MPBS) and MPBS alone (as coating control) were incubated at room temperature for 1 hour. For detection, 100 µl of the HRP-conjugated detection antibodies in MPBS (ATROSAB: goat anti-human IgG, Fab specific [Sigma Aldrich] diluted 1∶5,000; Htr-9: goat anti-murine IgG, Fc specific [Sigma Aldrich], diluted 1∶1,000; coating control: goat anti-human IgG, Fc specific [Sigma Aldrich], diluted 1∶5,000) were used, prior to detection with 100 µl TMB substrate solution (3,5,3′,5′-tetramethylbenzidine). The reaction was stopped by the addition of 50 µl 1 M H_2_SO_4_ and the absorption at the wavelength of 450 nm was measured using the Infinite microtiter plate reader (Tecan I control). Between each step, the plates were washed 3 times with PBS, 0.005% Tween20 and twice with PBS.

### Competition ELISA

Coating, blocking, detection and wash steps were performed as described for ELISA. The samples for the competition were 100 µl 7 nM Htr-9 mixed with ATROSAB (1 µM–5.7 pM, diluted in MPBS) and MPBS alone (as coating control), applied to the plates at room temperature for 1 hour.

### C1q and FcγR Binding ELISA

100 µl of 1 µM ATROSAB or 1 µM Trastuzumab were used for the coating of microtiter plates and incubated at 4°C overnight. The samples containing 100 µl C1q (Complement C1q, Human, Calbiochem), FcγRI (recombinant human CD64/FCHR1A) or FcγRIII (recombinant human Fc gamma receptor IIIA/CD16a) were titrated from 1000 nM or 500 nM to 3.8 pM (diluted in MPBS). Bound proteins were detected using the respective HRP-conjugated detection antibodies in 2% MPBS (C1q: Sheep polyclonal to C1q (HRP) [abcam] diluted 1∶100; FcγRI, FcγRIII: Anti-His-HRP [Roth], diluted 1∶1,000; coating control: goat anti-human IgG, Fc specific [Sigma Aldrich], diluted 1∶5,000). Blocking, washing and detection procedures were performed as described.

### Complement-dependent Cytolysis (CDC)

Kym-1 target cells (JCRB, art. # JCRB0627) bearing TNFR-1 were labeled with cell-permeant calcein (Invitrogen, Germany) for 30 minutes at 1 µM final calcein concentration in assay medium (RPMI, Thermo Fisher Scientific, supplemented with 2% FBS, Moregate). Cells were washed twice in assay medium and then seeded onto 96-well microtiter plates (1×10^4^ cells/well). ATROSAB was added at a maximal concentration of 600 µg/ml (and 1∶10 dilutions thereof). Dilutions were carried out in assay medium diluted with formulation buffer of ATROSAB (25 mM histidine, 102 mM NaCl, 26 mM trehalose, 0.04% Tween 20, pH 6.2) equivalent to the highest ATROSAB concentration, thus, each well contained the same amount of ATROSAB formulation buffer, compensating for potential buffer effects on cell viability *per se*. Then, human serum (Blood Donation Center SRK Basel; www.blutspende-basel.ch/) was added to the cell-antibody mixture and release of calcein was measured after 2 h. Three different dilutions (1∶2, 1∶4, and 1∶8) of each of the two serum batches were carried out in medium supplemented with 10% FBS prior to addition of serum to the cell-antibody mixture. A commercially available anti-CD20 antibody (Rituximab), which is known to induce cell lysis by CDC in CD20-expressing cells, was used at 12-fold lower concentration (50 µg/ml and 1∶10 dilutions thereof) as compared to ATROSAB, and served as positive control. The target cell line for this antibody was DOHH-2 (human B-cell lymphoma cell line, DSMZ, ACC-47). As controls served cells treated with 0.5% final concentration of Triton X-100 (maximal release of calcein = 100% relative cell lysis/positive control) or the medium-buffer mixture (containing buffer equivalent to the highest antibody concentration), representing the condition of spontaneous calcein release (i.e. 0% relative cell lysis/negative control). Statistical analysis was performed with Student’s t-test.

### Antibody-dependent Cellular Cytotoxicity (ADCC)

PBMCs were purified from fresh human whole blood (blood donation centre, Basel) as source of NK cells. Kym-1 target cells bearing the ATROSAB target receptor TNFR-1 were labeled for 30 minutes with calcein (1 µM final calcein concentration in assay medium, see CDC) and subsequently washed prior to seeding onto 96-well microtiter plates (1×10^4^ cells/well). ATROSAB was added at a maximal concentration of 600 µg/ml (and 1∶5 dilutions thereof). Dilutions were carried out in medium diluted with reconstitution buffer of ATROSAB equivalent to the highest ATROSAB concentration (600 µg/ml). Then, the PBMC effector cell fraction was added to the cell-antibody mixture at the indicated effector-to-target (E:T) cell ratios and calcein release was measured after 4 h of incubation. A commercially available anti-CD20 antibody known to induce cell lysis by ADCC in CD20-expressing cells was used at 12-fold lower concentration (50 µg/ml and 1∶5 dilutions thereof) as compared to ATROSAB and served as positive control (target cell line DOHH-2). As controls, cells treated with 0.5% final concentration of Triton X-100 (maximal release of calcein = 100% relative cell lysis/positive control) or the medium-buffer mixture (containing buffer equivalent to the highest antibody concentration) was added to cells instead of antibody (spontaneous release of calcein = 0% relative cell lysis/negative control). Statistical analysis was performed with Student’s t-test.

## References

[1] Schall TJ, Lewis M, Koller KJ, Lee A, Rice GC, et al (1990) Molecular cloning and expression of a receptor for human tumor necrosis factor. Cell 61: 361–370. 2158863 10.1016/0092-8674(90)90816-w

[2] Loetscher H, Pan YC, Lahm HW, Gentz R, Brockhaus M, et al (1990) Molecular cloning and expression of the human 55 kd tumor necrosis factor receptor. Cell 61: 351–359. 2158862 10.1016/0092-8674(90)90815-v

[3] Himmler A, Maurer-Fogy I, Krönke M, Scheurich P, Pfizenmaier K, et al (1990) Molecular cloning and expression of human and rat tumor necrosis factor receptor chain (p60) and its soluble derivative, tumor necrosis factor-binding protein. DNA Cell Biol 9: 705–715. 1702293 10.1089/dna.1990.9.705

[4] Locksley RM, Killeen N, Lenardo MJ (2001) The TNF and TNF receptor superfamilies: integrating mammalian biology. Cell 104: 487–501. 11239407 10.1016/s0092-8674(01)00237-9

[5] Cabal-Hierro L, Lazo PS (2012) Signal transduction by tumor necrosis factor receptors. Cell Signal. 24: 1297–1305. 10.1016/j.cellsig.2012.02.006 22374304

[6] Wajant H, Pfizenmaier K, Scheurich P (2003) Tumor necrosis factor signaling. Cell Death Differ 10: 45–65. 12655295 10.1038/sj.cdd.4401189

[7] Grell M, Douni E, Wajant H, Löhden M, Clauss M, et al (1995) The transmembrane form of tumor necrosis factor is the prime activating ligand of the 80 kDa tumor necrosis factor receptor. Cell 83: 793–802. 8521496 10.1016/0092-8674(95)90192-2

[8] Fontaine V, Mohand-Said S, Hanoteau N, Fuchs C, Pfizenmaier K, et al (2002) Neurodegenerative and neuroprotective effects of tumor Necrosis factor (TNF) in retinal ischemia: opposite roles of TNF receptor 1 and TNF receptor 2. J Neurosci 22: RC216. 11917000 10.1523/JNEUROSCI.22-07-j0001.2002 PMC6758303

[9] Goukassian DA, Qin G, Dolan C, Murayama T, Silver M, et al (2007) Tumor necrosis factor-alpha receptor p75 is required in ischemia-induced neovascularization. Circulation 115: 752–762. 17261656 10.1161/CIRCULATIONAHA.106.647255

[10] Tracey D, Klareskog L, Sasso EH, Salfeld JG, Tak PP (2008) Tumor necrosis factor antagonist mechanisms of action: a comprehensive review. Pharmacol Ther 117: 244–279. 18155297 10.1016/j.pharmthera.2007.10.001

[11] Kontermann RE, Scheurich P, Pfizenmaier K (2009) Antagonists of TNF action -clinical experience and new developments. Expert Opin Drug Discov 4: 279–292. 23489126 10.1517/17460440902785167

[12] Bongartz T, Sutton AJ, Sweeting MJ, Buchan I, Matteson EL, et al (2006) Anti-TNF antibody therapy in rheumatoid arthritis and the risk of serious infections and malignancies: systematic review and meta-analysis of rare harmful effects in randomized controlled trials. JAMA 295: 2275–2285. 16705109 10.1001/jama.295.19.2275

[13] Desai SB, Furst DE (2006) Problems encountered during anti-tumour necrosis factor therapy. Best Pract Res Clin Rheumatol. 20: 757–790. 10.1016/j.berh.2006.06.002 16979537

[14] Wallis RS (2008) Tumour necrosis factor antagonists: structure, function, and tuberculosis risks. Lancet Infect Dis 8: 601–611. 18922482 10.1016/S1473-3099(08)70227-5

[15] Rosenblum H, Amital H (2011) Anti-TNF therapy: safety aspects of taking the risk. Autoimmun Rev 10: 563–568. 21570495 10.1016/j.autrev.2011.04.010

[16] Zidi I, Bouaziz A, Mnif W, Bartegi A, Ben Amor N (2011) Golimumab and malignancies: true or false association? Med Oncol 28: 641–648. 20373059 10.1007/s12032-010-9490-7

[17] Shakoor N, Michalska M, Harris CA, Block JA (2002) Drug-induced systemic lupus erythematosus associated with etanercept therapy. Lancet 359: 579–580. 11867114 10.1016/S0140-6736(02)07714-0

[18] de Gannes GC, Ghoreishi M, Pope J, Russell A, Bell D, et al (2007) Psoriasis and pustular dermatitis triggered by TNF-α inhibitors in patients with rheumatologic conditions. Arch Dermatol 143: 223–231. 17310002 10.1001/archderm.143.2.223

[19] Ramos-Casals M, Brito-Zerón P, Muñoz S, Soria N, Galiana D, et al (2007) Autoimmune diseases induced by TNF-targeted therapies: analysis of 233 cases. Medicine (Baltimore) 86: 242–251. 17632266 10.1097/MD.0b013e3181441a68

[20] Tack CJ, Kleijwegt FS, Van Riel PL, Roep BO (2009) Development of type 1 diabetes in a patient treated with anti-TNF-alpha therapy for active rheumatoid arthritis. Diabetologia 52: 1442–1444. 19440690 10.1007/s00125-009-1381-0 PMC2688610

[21] Arnett HA, Mason J, Marino M, Suzuki K, Matsushima GK, et al (2001) TNF alpha promotes proliferation of oligodendrocyte progenitors and remyelination. Nat. Neurosci 4: 1116–1122. 10.1038/nn738 11600888

[22] Kassiotis G, Kollias G (2001) Uncoupling the proinflammatory from the immunosuppressive properties of tumor necrosis factor (TNF) at the p55 TNF receptor level: implications for pathogenesis and therapy of autoimmune demyelination. J. Exp. Med. 193, 427–434. 10.1084/jem.193.4.427 PMC2195909 11181695

[23] Van Hauwermeiren F, Vandenbroucke RE, Libert C (2011) Treatment of TNF mediated diseases by selective inhibition of soluble TNF or TNFR1. Cytokine Growth Factor Rev 22: 311–319. 21962830 10.1016/j.cytogfr.2011.09.004

[24] Shibata H, Yoshioka Y, Ohkawa A, Abe Y, Nomura T, et al (2008) The therapeutic effect of TNFR1-selective antagonistic mutant TNF-alpha in murine hepatitis models. Cytokine 44: 229–233. 18815054 10.1016/j.cyto.2008.07.003

[25] Shibata H, Yoshioka Y, Abe Y, Ohkawa A, Nomura T, et al (2009) The treatment of established murine collagen-induced arthritis with a TNFR1-selective antagonistic mutant TNF. Biomaterials 30: 6638–6647. 19765818 10.1016/j.biomaterials.2009.08.041

[26] Nomura T, Abe Y, Kamada H, Shibata H, Kayamuro H, et al (2011) Therapeutic effect of PEGylated TNFR1-seletive antagonistic mutant TNF in experimental autoimmune encephalomyelitis mice. J Control Release 149: 8–14. 20036293 10.1016/j.jconrel.2009.12.015

[27] Kitagaki M, Isoda K, Kamada H, Kobayashi T, Tsunoda S, et al (2012) Novel TNF-α receptor 1 antagonist treatment attenuates arterial inflammation and intimal hyperplasia in mice. J Atheroscler Thromb 19: 36–40. 22146239 10.5551/jat.9746

[28] Zalevsky J, Secher T, Ezhevsky SA, Janot L, Steed PM, et al (2007) Dominant-negative inhibitors of soluble TNF attenuate experimental arthritis without suppressing innate immunity to infection. J Immunol 179: 1872–1883. 17641054 10.4049/jimmunol.179.3.1872

[29] Olleros ML, Vesin D, Lambou AF, Janssens JP, Ryffel B, et al (2009) Dominant-negative tumor necrosis factor protects from Mycobacterium bovis Bacillus Calmette Guérin (BCG) and endotoxin-induced liver injury without compromising host immunity to BCG and Mycobacterium tuberculosis. J Infect Dis 199: 1053–1063. 19222369 10.1086/597204

[30] Perrier C, de Hertogh G, Cremer J, Vermeire S, Rutgeerts P, et al (2013) Neutralization of membrane TNF, but not soluble TNF, is crucial for the treatment of experimental colitis. Inflamm Bowel Dis 19: 246–253. 22649027 10.1002/ibd.23023

[31] Arntz OJ, Geurts J, Veenbergen S, Bennink MB, van den Brand BT, et al (2010) A crucial role for tumor necrosis factor receptor 1 in synovial lining cells and the reticuloendothelial system in mediating experimental arthritis. Arthritis Res Ther 12: R61. 20370892 10.1186/ar2974 PMC2888212

[32] Huang XW, Yang J, Dragovic AF, Zhang H, Lawrence TS, et al (2006) Antisense oligonucleotide inhibition of tumor necrosis factor receptor 1 protects the liver from radiation-induced apoptosis. Clin Cancer Res 12: 2849–2855. 16675580 10.1158/1078-0432.CCR-06-0360

[33] Thoma B, Grell M, Pfizenmaier K, Scheurich P (1990) Identification of a 60-kD tumor necrosis factor (TNF) receptor as the major signal transducing component in TNF responses. J Exp Med 172: 1019–1023. 2170559 10.1084/jem.172.4.1019 PMC2188608

[34] Kruppa G, Thoma B, Machleidt T, Wiegmann K, Krönke M (1992) Inhibition of tumor necrosis factor (TNF)-mediated NF-kappa B activation by selective blockade of the human 55-kDa TNF receptor. J Immunol 148: 3152–3157. 1315830

[35] Moosmayer D, Dübel S, Brocks B, Watzka H, Hampp C, et al (1995) A single-chain TNF receptor antagonist is an effective inhibitor of TNF mediated cytotoxicity. Ther Immunol 2: 31–40. 7553069

[36] Kontermann RE, Münkel S, Neumeyer J, Müller D, Branschädel M, et al (2008) A humanized tumor necrosis factor receptor 1 (TNFR1)-specific antagonistic antibody for selective inhibition of tumor necrosis factor (TNF) action. J Immunother 31: 225–234. 18317365 10.1097/CJI.0b013e31816a88f9

[37] Zettlitz KA, Lorenz V, Landauer K, Münkel S, Herrmann A, et al (2010) ATROSAB, a humanized antagonistic anti-tumor necrosis factor receptor one-specific antibody. MAbs 2: 639–647. 20935477 10.4161/mabs.2.6.13583 PMC3011218

[38] Armour KL, Clark MR, Hadley AG, Williamson LM (1999) Recombinant human IgG molecules lacking Fcγ receptor I binding and monocyte triggering activities. Eur J Immunol 29: 2613–2624. 10458776 10.1002/(SICI)1521-4141(199908)29:08<2613::AID-IMMU2613>3.0.CO;2-J

[39] Bruhns P, Iannascoli B, England P, Mancardi DA, Fernandez N, et al (2009) Specificity and affinity of human Fcγ receptors and their polymorphic variants for human IgG subclasses. Blood 113: 3716–3725. 19018092 10.1182/blood-2008-09-179754

[40] Moore GL, Chen H, Karki S, Lazar GA (2010) Engineered Fc variant antibodies with enhanced ability to recruit complement and mediate effector functions. MAbs 2: 181–189. 20150767 10.4161/mabs.2.2.11158 PMC2840237

[41] Grell M, Scheurich P, Meager A, Pfizenmaier K (1993) TR60 and TR80 tumor necrosis factor (TNF)-receptors can independently mediate cytolysis. Lymphokine Cytokine Res 12: 143–148. 8394147

[42] Brockhaus M, Schoenfeld HJ, Schlaeger EJ, Hunziker W, Lesslauer W, et al (1990) Identification of two types of tumor necrosis factor receptors on human cell lines by monoclonal antibodies. Proc Natl Acad Sci USA 87: 3127–3131. 2158104 10.1073/pnas.87.8.3127 PMC53847

[43] Espevik T, Brockhaus M, Loetscher H, Nonstad U, Shalaby R (1990) Characterization of binding and biological effects of monoclonal antibodies against a human tumor necrosis factor receptor. J Exp Med 171: 415–426. 1689365 10.1084/jem.171.2.415 PMC2187730

[44] Banner DW, D’Arcy A, Janes W, Gentz R, Schoenfeld HJ, et al (1993) Crystal structure of the soluble human 55 kd TNF receptor-human TNFb complex: implications for TNF receptor activation. Cell 73: 431–445. 8387891 10.1016/0092-8674(93)90132-a

[45] Li F, Ravetch JV (2012) A general requirement of FcγRIIB co-engagement of agonistic anti-TNFR antibodies. Cell Cycle 11: 3343–3344. 22918247 10.4161/cc.21842 PMC3466534

[46] Nygren H, Czerkinsky C, Stenberg M (1985) Dissociation of antibodies bound to surface-immobilized antigen. J Immunol Methods 85: 87–95. 3908564 10.1016/0022-1759(85)90276-5

[47] Kaufman EN, Jain RK (1992) Effect of bivalent interaction upon apparent antibody affinity: experimental confirmation of theory using fluorescence photobleaching and implications for antibody binding assays. Cancer Res 52: 4157–4167. 1638531

[48] Grell M, Wajant H, Zimmermann G, Scheurich P (1998) The type 1 receptor (CD120a) is the high affinity receptor for soluble tumor necrosis factor. Proc Natl Acad Sci USA 95: 570–575. 9435233 10.1073/pnas.95.2.570 PMC18461

[49] Chan FK, Chun HJ, Zheng L, Siegel RM, Bui KL, et al (2000) A domain in TNF receptors that mediates ligand-independent receptor assembly and signaling. Science 288: 2351–2354. 10875917 10.1126/science.288.5475.2351

[50] Legler DF, Micheau O, Doucey MA, Tschopp J, Bron C (2003) Recruitment of TNF receptor 1 to lipid rafts is essential for TNFα-mediated NFκB activation. Immunity 18: 655–664. 12753742 10.1016/s1074-7613(03)00092-x

[51] Ranzinger J, Krippner-Heidenreich A, Haraszti T, Bock E, Tepperink J, et al (2009) Nanoscale arrangement of apoptotic ligands reveals a demand for a minimal lateral distance for efficient death receptor activation. Nano Lett 9: 4240–4245. 19772290 10.1021/nl902429b PMC2905624

[52] Gerken M, Krippner-Heidenreich A, Steinert S, Willi S, Neugart F, et al (2010) Fluorescence correlation spectroscopy reveals topological segregation of the two tumor necrosis factor membrane receptors. Biochim Biophys Acta 1798: 1081–1089. 20188063 10.1016/j.bbamem.2010.02.021

[53] Branschädel M, Aird A, Zappe A, Tietz C, Krippner-Heidenreich A, et al (2010) Dual function of cysteine rich domain (CRD) 1 of TNF receptor type 1: conformational stabilization of CRD2 and control of receptor responsiveness. Cell Signal 22: 404–414. 19879354 10.1016/j.cellsig.2009.10.011

[54] Winkel C, Neumann S, Surulescu C, Scheurich P (2012) A minimal mathematical model for the initial molecular interactions of death receptor signalling. Math Biosci Eng 9: 663–683. 22881031 10.3934/mbe.2012.9.663

[55] Krippner-Heidenreich A, Tübing F, Bryde S, Willi S, Zimmermann G, et al (2002) Control of receptor-induced signaling complex formation by the kinetics of ligand/receptor interaction. J Biol Chem 277: 44155–44163. 12215450 10.1074/jbc.M207399200

[56] Lewis AK, Valley CC, Sachs JN (2012) TNFR1 signaling is associated with backbone conformational changes of receptor dimers consistent with overactivation in the R92Q TRAPS mutant. Biochemistry 551: 6546–6555. 10.1021/bi3006626 22799488

[57] Telliez JB, Xu GY, Woronicz JD, Hsu S, Wu JL, et al (2000) Mutational analysis and NMR studies of the death domain of the tumor necrosis factor receptor-1. J Mol Biol 300: 1323–1333. 10903872 10.1006/jmbi.2000.3899

[58] Sukits SF, Lin LL, Hsu S, Malakian K, Powers R, et al (2001) Solution structure of the tumor necrosis factor receptor-1 death domain. J Mol Biol 310: 895–906. 11453696 10.1006/jmbi.2001.4790

[59] Calmon-Hamaty F, Combe B, Hahne M, Morel J (2011) Lymphotoxin α revisted: general features and implications in rheumatoid arthritis. Arthritis Res 13: 232. 10.1186/ar3376 PMC3239340 21861866

[60] Ruddle NH, Steinman L (1990) Lymphotoxin and tumor necrosis factor-alpha production by myelin basic protein-specific T cell clones correlates with encephalitogenicity. Int Immunol 2: 539–544. 1707660 10.1093/intimm/2.6.539

[61] Suen WE, Bergman CM, Hjelmström P, Ruddle NH (1997) A critical role of lymphotoxin in experimental allergic encephalomyelitis. J Exp Med 186: 1233–1240. 9334362 10.1084/jem.186.8.1233 PMC2199100

[62] Chiang EY, Kolumam GA, Yu X, Francesco M, Ivelja S, et al (2009) Targeted depletion of lymphotoxin-α-expressing TH1 and TH17 cells inhibits autoimmune disease. Nat Med 15: 766–773. 19561618 10.1038/nm.1984

[63] Buch MH, Conaghan PG, Quinn MA, Bingham SJ, Veale D, et al (2004) True infliximab resistance in rheumatoid arthritis: a role for lymphotoxin α? Ann Rheum Dis 63: 1344–1346. 15033655 10.1136/ard.2003.014878 PMC1754777

[64] Grell M, Zimmermann G, Hülser D, Pfizenmaier K, Scheurich P (1994) TNF receptors TR60 and TR80 can mediate apoptosis via induction of distinct signal pathways. J Immunol 153: 1963–1972. 8051401

[65] Carter PH, Scherle PA, Muckelbauer JA, Voss ME, Liu RQ, et al (2001) Photochemically enhanced binding of small molecules to the tumor necrosis factor receptor-1 inhibits the binding of TNF-a. Proc Natl Acad Sci USA 98: 11879–11884. 11592999 10.1073/pnas.211178398 PMC59736

